# Supplementary material for: Novel Driver Strength Index highlights important cancer genes in TCGA PanCanAtlas patients
Source: PeerJ. 2022 Aug 11;10:e13860. doi: 10.7717/peerj.13860 (PMC9375969; doi:10.7717/peerj.13860)
Supplement: Supplemental Information 2 [file peerj-10-13860-s002.zip › COHORTS/genes plots/detailed/2021_8_16_14_9_distribution_events_detailed_males_7.pdf]

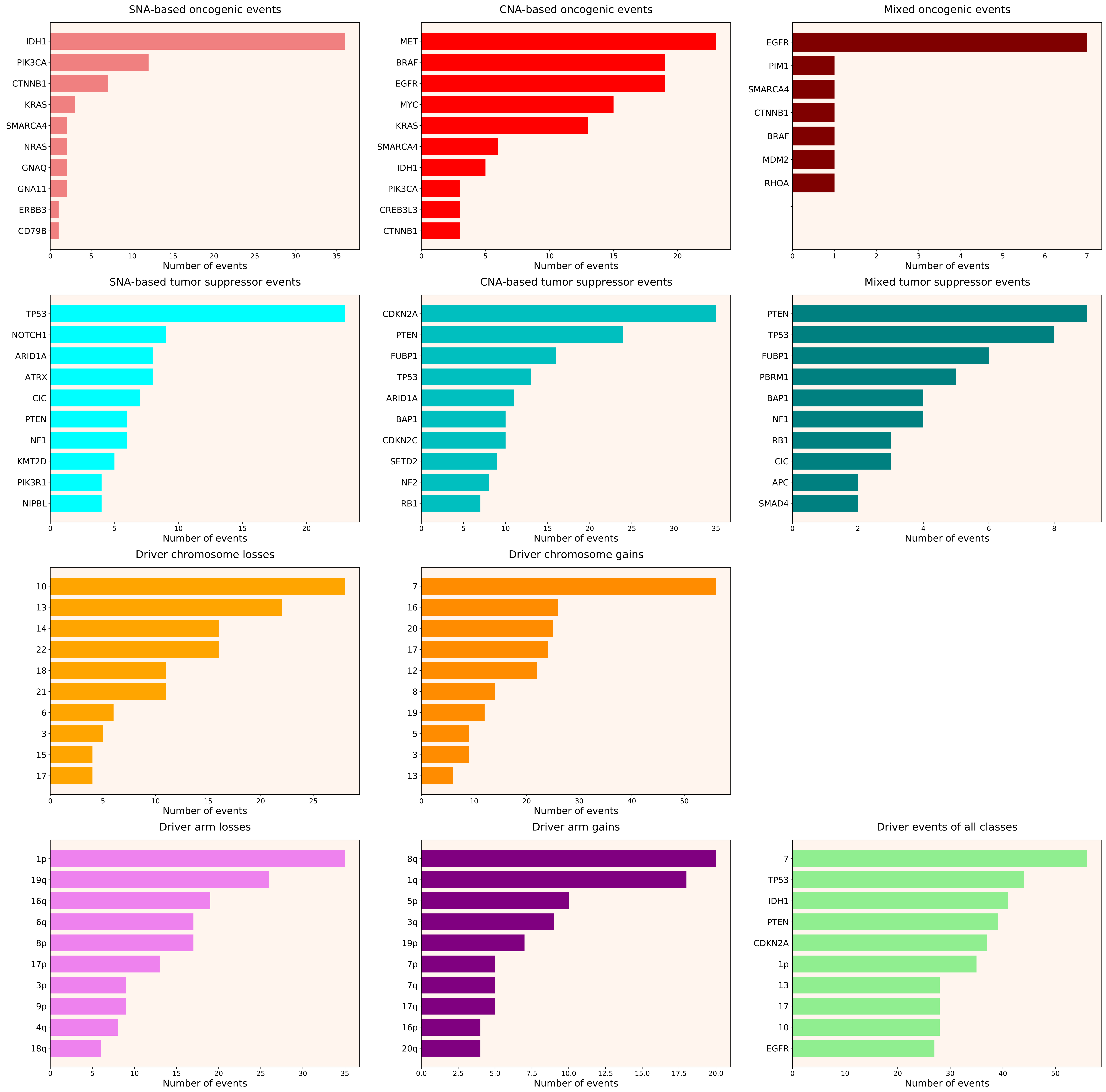

SNA-based oncogenic events
IDH1
PIK3CA
CTNNB1
KRAS
SMARCA4
NRAS
GNAQ
GNA11
ERBB3
CD79B
0 5 10 15 20 25 30 35
Number of events
CNA-based oncogenic events
MET
BRAF
EGFR
MYC
KRAS
SMARCA4
IDH1
PIK3CA
CREB3L3
CTNNB1
0 5 10 15 20
Number of events
Mixed oncogenic events
EGFR
PIM1
SMARCA4
CTNNB1
BRAF
MDM2
RHOA
0 1 2 3 4 5 6 7
Number of events
SNA-based tumor suppressor events
TP53
NOTCH1
ARID1A
ATRX
CIC
PTEN
NF1
KMT2D
PIK3R1
NIPBL
0 5 10 15 20
Number of events
CNA-based tumor suppressor events
CDKN2A
PTEN
FUBP1
TP53
ARID1A
BAP1
CDKN2C
SETD2
NF2
RB1
0 5 10 15 20 25 30 35
Number of events
Mixed tumor suppressor events
PTEN
TP53
FUBP1
PBRM1
BAP1
NF1
RB1
CIC
APC
SMAD4
0 2 4 6 8
Number of events
Driver chromosome losses
10
13
14
22
18
21
6
3
15
17
0 5 10 15 20 25
Number of events
Driver chromosome gains
7
16
20
17
12
8
19
5
3
13
0 10 20 30 40 50
Number of events
Driver arm losses
1p
19q
16q
6q
8p
17p
3p
9p
4q
18q
0 5 10 15 20 25 30 35
Number of events
Driver arm gains
8q
1q
5p
3q
19p
7p
7q
17q
16p
20q
0.0 2.5 5.0 7.5 10.0 12.5 15.0 17.5 20.0
Number of events
Driver events of all classes
7
TP53
IDH1
PTEN
CDKN2A
1p
13
17
10
EGFR
0 10 20 30 40 50
Number of events
